# Censored data considerations and analytical approaches for salivary bioscience data

**DOI:** 10.1016/j.psyneuen.2021.105274

**Published:** 2021-05-17

**Authors:** Hedyeh Ahmadi, Douglas A. Granger, Katrina R. Hamilton, Clancy Blair, Jenna L. Riis

**Affiliations:** a Institute for Interdisciplinary Salivary Bioscience Research, University of California, Irvine, CA, USA; b Department of Psychological Science, University of California, Irvine, CA, USA; c Department of Acute and Chronic Care, Johns Hopkins University School of Nursing, Baltimore, MD, USA; d Department of Pediatrics, Johns Hopkins University School of Medicine, Baltimore, MD, USA; e Salivary Bioscience Laboratory and Department of Psychology, University of Nebraska, Lincoln, NE, USA; f Department of Population Health and Department of Applied Psychology, New York University, New York, NY, USA

**Keywords:** Censored data, Tobit regression Saliva, C-reactive protein, Statistical analysis

## Abstract

Left censoring in salivary bioscience data occurs when salivary analyte determinations fall below the lower limit of an assay’s measurement range. Conventional statistical approaches for addressing censored values (i.e., recoding as missing, substituting or extrapolating values) may introduce systematic bias. While specialized censored data statistical approaches (i.e., Maximum Likelihood Estimation, Regression on Ordered Statistics, Kaplan-Meier, and general Tobit regression) are available, these methods are rarely implemented in biobehavioral studies that examine salivary biomeasures, and their application to salivary data analysis may be hindered by their sensitivity to skewed data distributions, outliers, and sample size. This study compares descriptive statistics, correlation coefficients, and regression parameter estimates generated via conventional and specialized censored data approaches using salivary C-reactive protein data. We assess differences in statistical estimates across approach and across two levels of censoring (9% and 15%) and examine the sensitivity of our results to sample size. Overall, findings were similar across conventional and censored data approaches, but the implementation of specialized censored data approaches was more efficient (i.e., required little manipulations to the raw analyte data) and appropriate. Based on our review of the findings, we outline preliminary recommendations to enable investigators to more efficiently and effectively reduce statistical bias when working with left-censored salivary biomeasure data.

## 1. Introduction

Researchers across a wide range of disciplines measure analytes in saliva to index the activity, reactivity, and regulation of physiologic systems (see Granger and Taylor (2020) for review). Dispatches from the cutting edge of this effort reveal unique statistical challenges (e.g., Riis et al., 2020). It is common for measurements from oral fluids to show skewed or atypical distributions. For instance, the levels of disease-specific antibodies and antigens, hormones, cytokines, and environmental chemicals and metals in saliva may be expectedly low or below assay detection limits in the general population. In this paper, we address a common, but often overlooked, measurement and statistical complication of salivary biomeasure data – the issue of left-censored data.

**Fig. 1.**
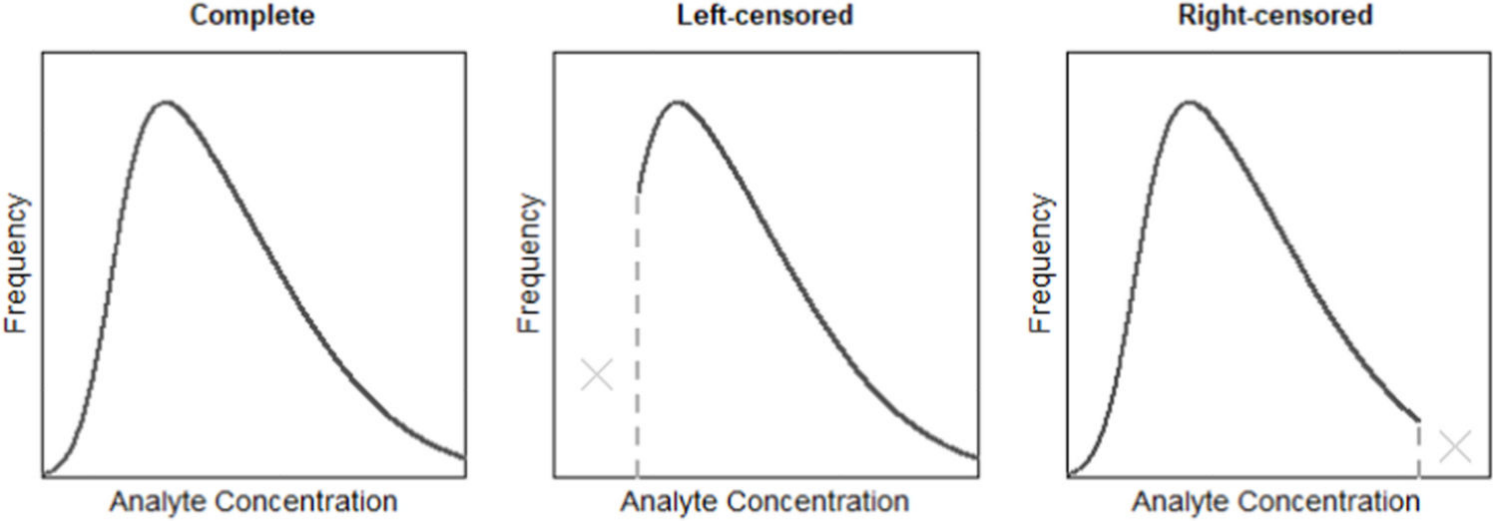
Left- and right-censored data distributions compared to complete data. For salivary analyte data, the dashed lines represent the assay’s lower (left-censored) and upper (right-censored) limits of measurement.

Censoring occurs when there is only partial information on quantitative observations in a dataset (Helsel, 2012). In salivary bioscience, censoring is the result of sample determinations falling outside the lower or upper limits of an assay’s measurement range (see Fig. 1; Helsel, 2005, 2012). Data are left-censored when values fall below the assay’s lowest measurable concentration (i.e., the lower limit of quantification, sensitivity, or detection). These data are often referred to as “non-detects” and may be included in laboratory reports as either missing or with a qualitative measurement (e.g., “_<_ the lowest limit”). Left censoring is common when levels of a salivary analyte are naturally very low or when an assay’s lower limit of measurement is inappropriately matched to the expected range of values. For analytes present at high levels in saliva, or for assays with inappropriately low upper limits of measurement, right censoring can present a problem. Right-censored data occur when analyte determinations are high relative to an assay’s scale of measurement, and this issue is most often resolved by retesting samples on dilution (Chard, 1990).

### 1.1. Conventional approaches to censored data

Our informal review of the literature suggests that scientists working with salivary analyte data have typically employed one of three approaches for handling left-censored values: 1) recoding them as missing; 2) substituting them with a constant such as the assay’s lower limit of measurement, half the assay’s lower limit, zero, or a value close to zero (to allow for logarithmic transformations of the data); or 3) extrapolating them based on the assay’s standard curve and dynamic range (Riis et al., 2020). After implementing one of these approaches, investigators can employ general statistical modeling techniques. While computationally simple, these approaches may introduce systematic bias. They also present problems with missingness, as censored values recoded to missing represent non-random missingness; and these values cannot be addressed with missing data approaches that assume missing at random or missing completely at random missingness mechanisms. Moreover, substituting censored values alters the data distribution and can bias analytic results. The degree of bias introduced by these conventional approaches likely varies by the sample size and percent of censored data (Helsel, 2006; Antweiler and Taylor, 2008; Tekindal et al., 2017; Canales et al., 2018).

### 1.2. Censored data approaches

Specialized statistical approaches for censored data have been used in other fields and can be applied in salivary bioscience studies. These approaches can be used to generate summary statistics (e.g., mean, median, and standard deviation estimates) for analytes with non-detect determinations and to estimate associations (e.g., correlation coefficients and regression parameters) between these analytes and other key variables of interest. Specialized censored data methods use information about the proportion or rank of the censored data within a given variable to generate descriptive statistics and model parameters. By using the partial information provided by censored data points, these approaches typically generate less-biased descriptive and parameter estimates for variables with censored cases.

#### 1.2.1. Descriptive statistics

Conventional approaches to calculating summary statistics for censored salivary biomeasure data either ignore (when censored data are recoded to missing) or mis-specify (when censored data are substituted or extrapolated) censored data. As an alternative to these approaches, investigators can use specialized censored data methods to calculate summary statistics for salivary analyte data with non-detect determinations. These specialized approaches include: Maximum Likelihood Estimation (MLE), Regression on Ordered Statistics (ROS), and Kaplan-Meier (K-M). MLE is a parametric method for estimating summary statistics of censored variables. However, it is not suitable for small sample sizes nor highly skewed data, and it is sensitive to outliers (Helsel, 2012). For smaller samples and variables with high percentages of censoring (≤ 80%), ROS is a more appropriate approach (Ofungwu, 2014). If variables have multiple censoring thresholds, summary statistics can be estimated via the K-M approach. K-M is not sensitive to sample size and is appropriate for variables with ≤ 50% censoring. (Ofungwu, 2014; Shoari and Dubé, 2018). The descriptive statistics generated via all three of these specialized approaches allow the investigator to assess summary statistics for salivary biomeasures using all the analyte data in their sample (i.e., using both censored and un-censored determinations) and for specific groups (e.g., stratified by sex). These summary statistics, and the estimated variability of these statistics, are more appropriate than those generated via conventional approaches (e.g., dropping censored cases and calculating summary statistics for the un-censored data only), because they account for the level of censoring in the data in their estimations.

#### 1.2.2. Bivariate associations

When assessing unadjusted associations between variables with censored data, the Spearman and Kendall’s correlations are rank-based (non-parametric) approaches that provide appropriate estimates. When implemented with censored data, substitution approaches should be employed prior to estimating bivariate associations, as these correlations require that all censored data points have a value. During the Spearman and Kendall’s correlation estimation procedures, all censored values receive the same rank, making these approaches more appropriate than conventional, parametric tests of association such as the Pearson’s correlation (Helsel, 2012). While both the Spearman and Kendall’s correlations are rank-based, the estimation procedure of the Kendall’s correlation, which is based on concordance/discordance of the rank scores, makes the Kendall’s correlation more appropriate when the sample size is small and when there are outliers or extreme data points in the sample.

#### 1.2.3. Adjusted relations via regression

Several regression modeling approaches are available to estimate adjusted associations between variables with censored values. While many of these methods have limited applicability in salivary bioscience (see Supplemental Materials), general Tobit regression offers a parametric censored regression approach that is well-suited for left-censored salivary bioscience data (e.g., Uh et al., 2008; Ford and Stowe, 2017). Its implementation parallels that of multivariable linear regression, and it can be used to assess associations between a censored outcome variable and one or more predictor variables. General Tobit regression also allows for log-normality assumptions, rather than the standard assumption of normality. The regression parameters estimated via general Tobit regression are generated using partial information provided by the censored data points, making these estimates more appropriate than those generated via the conventional approaches.

### 1.3. Present study

The impact of using conventional vs. specialized censored data statistical approaches on research findings has not been rigorously examined in salivary bioscience investigations. To address this gap, we compare results from conventional and censored data methods when used to examine salivary C-reactive protein (CRP) and its bivariate and adjusted associations with body mass index (BMI). We compare descriptive statistics, correlations, and regression parameter estimates generated using the substitution and deletion (i.e., recoding to missing) approaches to those generated using specialized, censored data statistical methods. Each adjusted regression model includes one continuous (BMI), one dichotomous (sex), and one three-level categorical (health status) independent variable. This allows us to explore differences in parameter estimates from conventional and specialized approaches associated with variable type. To assess how the censoring percentage affects the results across method, we compare these approaches under two levels of censoring- the true level of censoring and an artificially- inflated level of censoring that represents an alternate, realistic percentage of censoring for salivary CRP data.

## 2. Materials and methods

This study is a secondary analysis of data collected as part of the Family Life Project (FLP), a large-scale longitudinal investigation of child health and development in the context of rural poverty. The sample and study procedures, briefly described below, have been reported elsewhere (Vernon-Feagans and Cox, 2013; Camerota et al., 2020). Given the methodologic focus of this study, limited information is provided regarding the FLP sample and study procedures, and instead we focus on the analytic methods.

### 2.1. Participants and procedures

We examine data collected from child participants at the 12-year follow-up visit of the FLP study. The study sample includes participants who provided CRP data at this visit and whose saliva samples were assayed in the first round of testing (N = 635). The data used in this investigation were collected from July 2016 to September 2019. As part of the larger study procedures, participants provided an unstimulated whole saliva sample via passive drool and completed a series of surveys. Saliva samples were frozen to precipitate mucins and transported to the Institute for Interdisciplinary Salivary Bioscience Research for archiving at − 80 ^◦^C and assay.

### 2.2. Measures

#### 2.2.1. Salivary C-reactive protein

All saliva samples were assayed in duplicate for CRP using the Meso Scale Diagnostics (MSD) Human CRP (Vascular Injury Panel 2) V-Plex assay (Ref# K0080900). The manufacturer’s protocol was followed without modification. Samples were tested using a 5-fold dilution with a testing volume of 5 μL. CRP concentrations (pg/mL) were determined with the MSD Discovery Workbench Software (v. 4.0) using curve fit models (4-PL with a weighting function option of 1/y^2^). The assay range of sensitivity was 9.9–1,010,000 pg/mL. Intra- and inter-assay CVs were 3.40% and 10.15%, respectively.

To facilitate censored data analyses and the examination of how the level of censoring impacts the estimates generated by conventional and censored data analysis approaches, two dummy variables were created that coded for left censoring in salivary CRP. One dummy variable indexed true censoring due to the lower limit of sensitivity (LLOS) of the salivary assay (LLOS=9.9 pg/mL), and one indexed an artificially high level of censoring by using an inflated LLOS of 19.7 pg/mL. To prepare salivary CRP data for analyses with the conventional statistical approaches, six new salivary CRP variables (three per level of censoring) were created that recoded left-censored determinations as: 1) missing values (deletion approach); 2) half the LLOS (a common variant of the substitution approach); and 3) 0.01 pg/mL (a variant of the substitution approach that replaces censored data with a value close to zero).

#### 2.2.2. Body mass index percentile score

As a known correlate of CRP concentrations, participant BMI was examined as the main predictor of interest in our analyses. This allows us to assess changes in the estimated association between CRP and BMI across conventional and censored data analytic approaches. Participant BMI was calculated using standing height (inches) and weight (pounds) measurements assessed once by trained interviewers (shoes/heavy clothing removed), and age- and sex-specific BMI percentile scores were assigned to each participant using the Centers for Disease Control and Prevention’s guidelines (Kuczmarski et al., 2000).

#### 2.2.3. Covariates

In addition to BMI, we included participant sex (male/female) and overall health (a three-level categorical variable) as covariates in the regression models to examine differences in parameter estimates for continuous, dichotomous, and categorical variables across the conventional and censored data regression approaches. Participant overall health was self-reported on the day of assessment and responses were categorized on a scale from 1 (excellent) to 3 (fair/poor).

### 2.3. Statistical analyses

#### 2.3.1. Descriptive statistics and plots for salivary CRP

CRP data were examined using conventional and censored data visualization approaches, such as censored histograms, scatter plots, and boxplots. These plots were used to explore the distribution of CRP concentrations and compare raw and log-transformed salivary data. Summary statistics such as the mean, median, and standard deviation of salivary CRP were generated using the substitution and deletion approaches, and these results were compared to those estimated using censored data methods, including the MLE, ROS, and K-M approaches. The log-normality assumption of the MLE approach was checked using Q-Q plots. All plots and comparisons were examined using CRP data under both the true and artificially-inflated level of censoring (i.e., LLOS condition).

#### 2.3.2. Relationship between salivary CRP and BMI using conventional and censored data correlation and regression approaches

The distribution of censored and observed CRP determinations across the independent variables (i.e., BMI percentile score, participant health status, and sex) was assessed using visualizations and summary statistics under both LLOS conditions. Bivariate associations between BMI percentile scores and salivary CRP concentrations were estimated using Kendall’s, Spearman’s, and Pearson’s correlations. For each method, six correlation coefficients were estimated using data generated by the deletion and substitution approaches under both LLOS conditions. Six multivariable linear regressions examining associations between salivary CRP (outcome variable; log transformed) and BMI percentile score (main independent variable) adjusted for participant health status and sex were conducted using the salivary CRP data created via the substitution and deletion approaches under both LLOS conditions. The same relations were examined in two general Tobit regression models for salivary CRP (outcome variable) with the level of censoring specified as either 9.9 pg/mL or 19.7 pg/mL and assuming a log-normal distribution of CRP. Model parameter estimates and their standard errors were compared across conventional and censored data approaches and across the two LLOS conditions. Differences in parameter estimates across method and LLOS condition were explored and compared across variables (BMI percentile score, health status, sex) to assess the relative sensitivity of parameter estimates to analytic method across variable type (e.g., continuous vs. categorial). Model fit was examined using residual plots (e.g., scatter plots of fitted values versus model residuals and Q-Q plots) for all regression models. All analyses were conducted in R (R Core Team, 2019).

#### 2.3.3. Sensitivity analyses

To assess whether the size of our sample considerably affected the patterns of results from our models, we conducted a series of sensitivity analyses for all descriptive statistics, correlations, and regression analyses using a random subsample of 100 participants. To ensure these analyses provided an appropriate evaluation of the effect of sample size on our results, we randomly selected a subsample with the same levels of censoring under both LLOS conditions as seen in the full sample.

## 3. Results

### 3.1. Descriptive statistics and plots

The analytic sample had 622 participants (nine cases excluded due to CRP intra-assay CVs exceeding 20%, four cases excluded due to missing BMI, sex, or health data). At the time of the assessment, participants in the analytic sample were, on average 13.09 years old (median (SD) = 12.95 (0.46); range = 12.52–14.40). The sample was 51.13% females (n_female_= 318), and the majority of the participants were healthy (excellent and very good/good self-reported health = 18.49% and 67.85%, respectively). The BMI percentile scores ranged from 0.00 to 99.78 with a mean and median of 73.45 and 83.59, respectively (SD = 27.31).

**Fig. 2.**
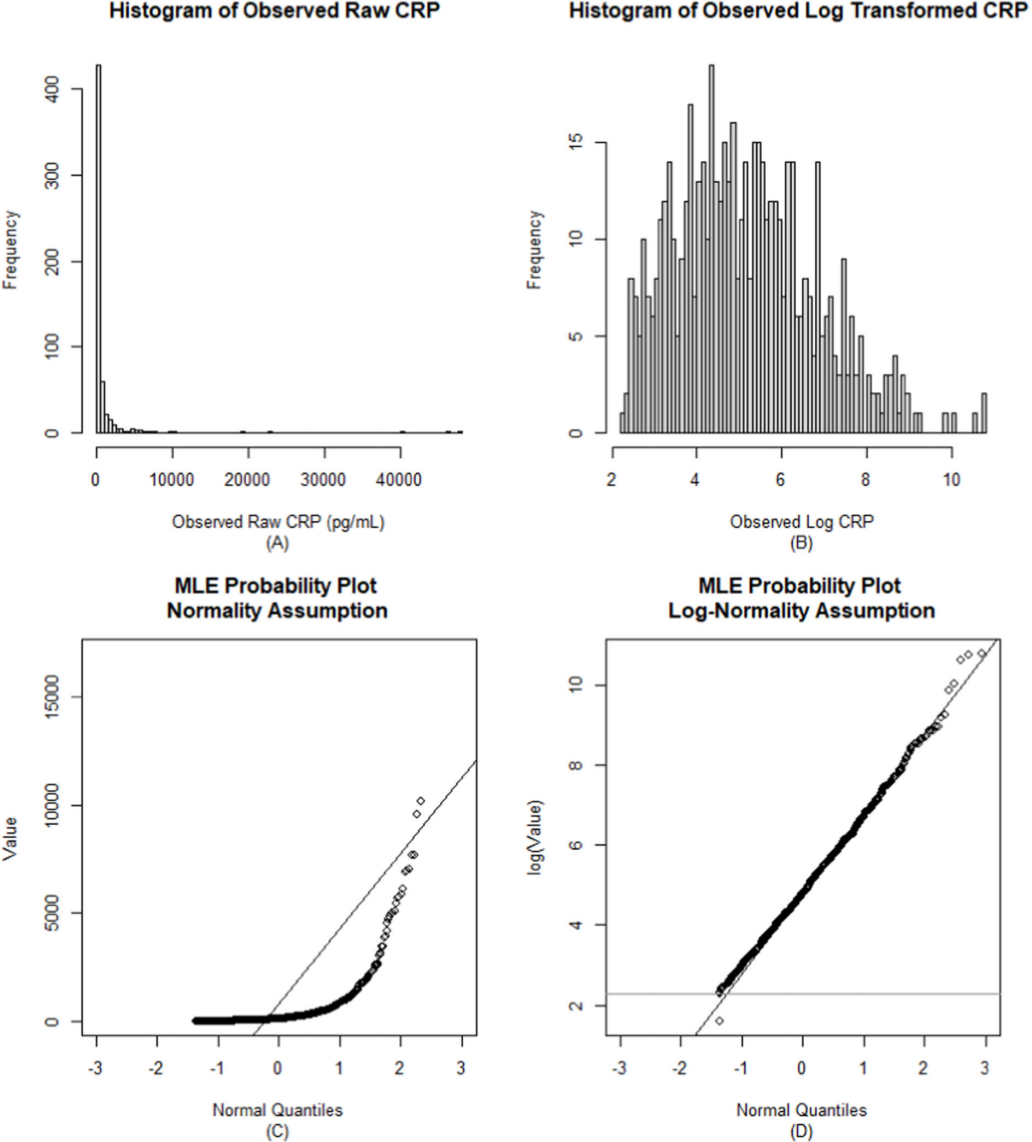
The distribution of salivary c-reactive protein (CRP) concentrations in early adolescence using the raw (left) and log-transformed (right) data. Panels A and B use the conventional deletion approach, and panels C and D use censored data visualization approaches. Note: N = 569 for panel A and B; N = 622 for panel C and D. These data are censored at the true lower limit of assay sensitivity (LLOS = 9.9 pg/mL; see Supplemental Figure A.1 for similar plots for the inflated LLOS of 19.7 pg/ mL). Panel C is a typical Q-Q plot with a normality assumption for right skewed data (i.e., concave up) showing the non-normal distribution of the salivary CRP data. Panel D assumes log-normality and shows strong improvement in the distribution of the data under this assumption. The horizontal gray line is drawn at the log of the LLOS (ln(9.9)). The point below this line in panel D represents the censored values.

The true level of censoring in the CRP data was 9% (n = 53-censored values), and all data were left-censored. Under the artificially-inflated LLOS, there was 15% left-censoring (n = 96 censored values). The range of observed CRP data prior to any modifications was 11.62–47,609.65 pg/mL. The distribution of the observed, raw salivary CRP data was skewed under both LLOS conditions (LLOS = 9.9 pg/mL: skew = 10.04, kurtosis = 114.14; LLOS = 19.7 pg/mL: skew = 9.67, kurtosis = 105.55). Log-transforming the salivary CRP data improved the normality of the data distribution under both LLOS conditions (Fig. 2 and A.1; log transformed CRP for LLOS = 9.9 pg/mL: skew = 0.53, kurtosis = 0.01; for LLOS = 19.7 pg/mL: skew = 0.68, kurtosis = 0.19), thus log-transformed salivary CRP data (or a log-normality assumption) were used in all regression models.

Summary statistics derived from conventional and censored data approaches for salivary CRP under the two LLOS conditions are shown in Table 1. Under both LLOS conditions, the mean, median, and standard deviation (SD) estimates generated using the K-M and ROS approaches were very similar to those generated under both substitution approaches. Mean and SD estimates from the MLE approach were considerably higher than those derived using the substitution, K-M, and ROS approaches, while median estimates were similar across these approaches. The deletion method resulted in the highest mean and median estimates for CRP. The substitution, K-M, and ROS approaches generated relatively stable descriptive statistics across LLOS condition, while the deletion and MLE estimates were more sensitive to the percent of censored data.

### 3.2. Relationship between salivary CRP and BMI using conventional and censored data correlation and regression approaches

**Fig. 3.**
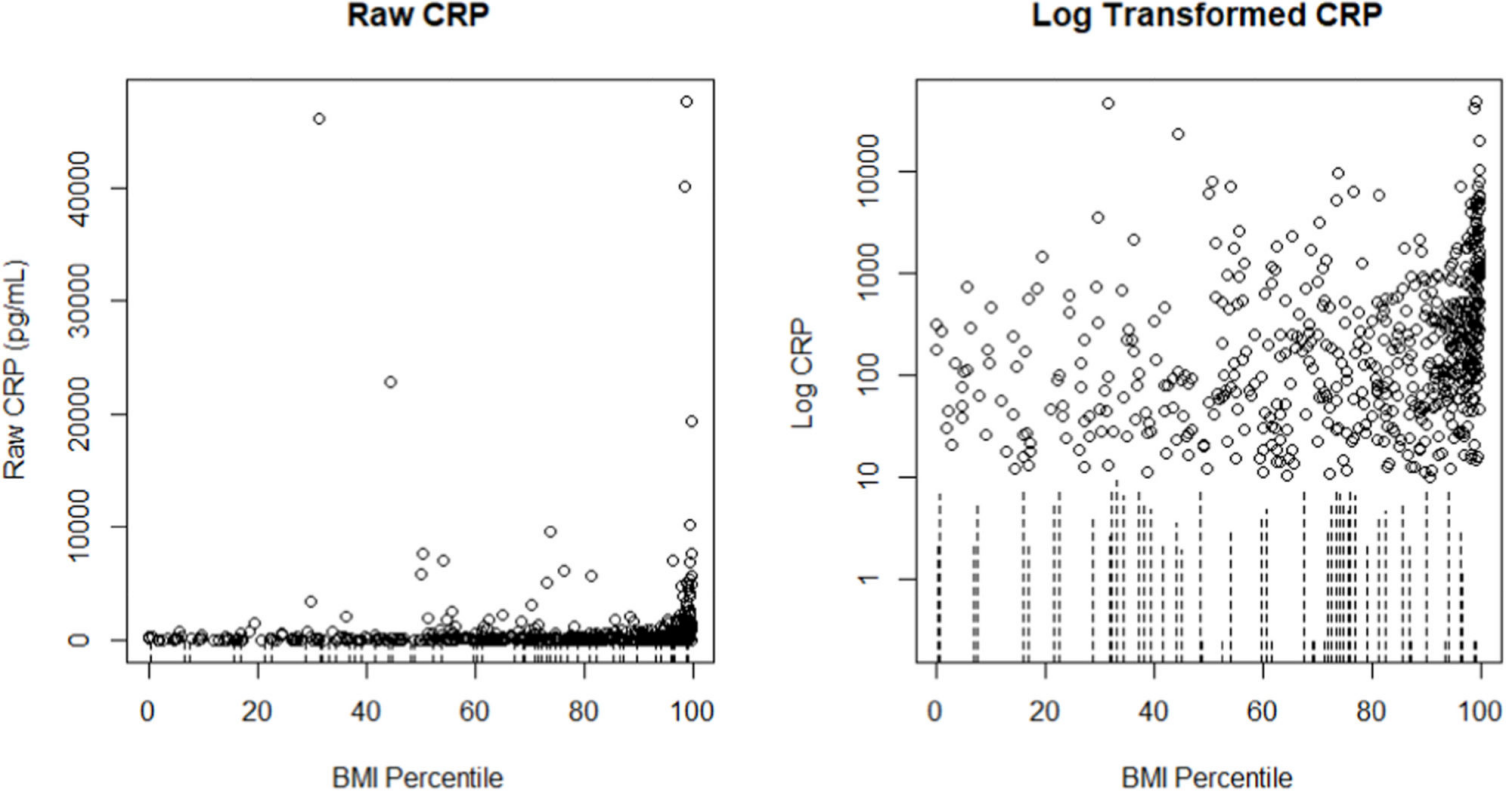
Censored data scatter plots for raw (left) and log-transformed (right) salivary c-reactive protein (CRP) concentrations from early adolescents (N = 622) showed minimal systematic patterns of censoring across body mass index (BMI) percentile score. Note: The average BMI percentile score for early adolescents with censored CRP determinations was 58.39 (median = 67.40; range = 0.26–98.08), and the average BMI percentile score for participants with observed CRP determinations was 74.85 (median = 85.57; range = 0.00–99.78).Observed concentrations of salivary CRP are plotted as individual points. Censored salivary CRP data are represented by dashed lines spanning from zero to the LLOS. These data are censored at the true lower limit of assay sensitivity (LLOS = 9.9 pg/mL; see Supplemental Fig. A.2 for similar plots for the artificially-inflated LLOS of 19.7 pg/mL).

Censored scatter plots examining the distribution of salivary CRP data by BMI percentile score (see Fig. 3 for LLOS of 9.9 pg/mL and Supplementary Figure A.2 for LLOS of 19.7 pg/mL) and censored boxplots of salivary CRP by sex and health status showed no observable, systematic clustering of censored salivary CRP data across these independent variables for either LLOS condition (see Supplemental Fig. A.3 and A.4).

Table 2 shows correlation coefficients for the association between salivary CRP and BMI percentile score estimated using conventional and censored data approaches under the two LLOS conditions. While all correlation coefficients across approach and LLOS condition were positive, only the censored data methods generated statistically significant estimates of association. The Spearman’s correlation coefficients were the strongest while the Pearson’s coefficients were the weakest. Correlation coefficients estimated using data generated via the deletion approach were consistently lower than those estimated using the substitution approaches. Regardless of approach, the percent of censoring had minimal impact on the estimated correlation coefficients.

Table 3 shows results from the multivariable linear regressions using the conventional and censored regression approaches under the true and artificially-inflated levels of censoring. Tobit regression coefficients can be interpreted similarly to coefficients in Ordinary Least Squares regression; the only caveat is that the linear effect is on the uncensored latent variable, not the observed outcome. At both 9% and 15% censoring, the parameter estimates for BMI percentile score are similar across all approaches (range of BMI percentile score $\hat{\beta }$= 0.01–0.04 across all approaches and LLOS conditions; Table 3). In general, the deletion approach produced the smallest parameter estimates of all the approaches while substitution with 0.01 pg/mL produced the largest estimations. The effect of approach on parameter estimates varied by variable with the maximum difference in coefficients across approach ranging from 38.56% to 409.72% in our models. The effect of approach on parameter estimates also varied by the percent of censored data, and this effect was not consistent across variables. The maximum difference in model coefficients across approach was larger for BMI percentile score and sex in the models with 15% censoring compared to those with 9% censoring, while the opposite was seen for the health status variables. Under both LLOS conditions, the results from models using the substitution with half the LLOS approach were the closest of the conventional approaches to the parameter estimates from the general Tobit model.

### 3.3. Model fit and diagnostics

For some of the regressions, Q-Q plots of the model residuals showed slight divergence from normality. Scatter plots of fitted values versus model residuals did not show heteroscedasticity problems in any of the models. Model residual plots from the linear regressions that used the substitution approaches showed slight clustering of residuals in two groups indicating potential problems with model fit.

### 3.4. Sensitivity analyses

The subsample of 100 participants used in sensitivity analyses consisted of 57% females (n_female_= 57) and the majority of the participants were healthy (excellent and very good/good self-reported health = 18.00% and 66.00%, respectively). The BMI percentile scores for this subsample ranged from 3.37 to 99.67 and the mean and median were 75.59 and 84.06, respectively (SD = 25.05).

Similar to the full sample, the true level of censoring in the CRP data for the subsample was 9% (n = 9 censored values) and all censored determinations were left-censored. Under the artificially-inflated LLOS of 19.7 pg/mL, there was 15% left-censoring (n = 15 censored values). The range of observed CRP data in this subsample prior to any modifications was 12.38–7027.62 pg/mL. The distribution of the observed, raw salivary CRP data was skewed under both LLOS conditions (LLOS = 9.9 pg/mL: skew = 2.53, kurtosis = 6.15; LLOS = 19.7 pg/mL: skew = 2.42, kurtosis = 5.53). Log-transforming the salivary CRP data improved the normality of the data distribution under both LLOS conditions (log transformed CRP for LLOS = 9.9 pg/mL: skew = 0.20, kurtosis = − 0.92; for LLOS = 19.7 pg/mL: skew = 0.28, kurtosis = − 0.94), thus log-transformed salivary CRP data (or a log- normality assumption) were used in all regression models.

#### 3.4.1. Descriptive statistics

The patterns of findings for the descriptive statistics of salivary CRP were similar for the subsample of 100 participants as those seen in the larger sample (Supplemental Table S1). However, the MLE median estimates from the subsample analyses diverged from those generated via the K-M, ROS, and substitution approaches more so than seen in the larger sample, and, unlike results from the larger sample, the MLE mean and SD estimates for the subsample were much larger than those generated via all other approaches, including the deletion approach (Supplemental Table S1).

#### 3.4.2. Correlation coefficients

Pearson’s correlation coefficients for the association between CRP and BMI percentile score were higher in the subsample (N = 100) than in the full sample (N = 622) (Supplemental Table S2). In contrast, the rank-based correlation coefficients were slightly weaker when assessed in the subsample than in the full sample (Supplemental Table S2). However, the pattern of statistically significant findings was the same in both samples (N = 100 and N = 622; Supplemental Table S2). In addition, the patterns of findings across approach and LLOS condition were similar in both samples (Supplemental Table S2).

#### 3.4.3. Regression parameters

When the regression analyses were conducted with the subsample of 100 participants, the parameter estimates for BMI percentile score were reduced, but they remained similar across all approaches and across LLOS condition (range of BMI percentile score $\hat{\beta }$ = 0.01–0.03 across all approaches and LLOS conditions; Supplemental Table S3). Similar to the results from the full sample, the effect of approach on parameter estimates varied by variable and by level of censoring. Also consistent with the results from the full sample, the results from subsample models using the substitution with half the LLOS approach were, in general, the closest of the conventional approaches to the parameter estimates from the general Tobit models.

## 4. Discussion

The primary goals of this work were to assist researchers in the application and interpretation of censored data methods with salivary biomeasure data, and to assess the impact of these approaches on study findings when compared to those generated using conventional methods. While our results show similar estimates across conventional and censored data methods, it is important to note some limitations of the conventional approaches employed.

Substituting censored data points with a single value (e.g., half the LLOS) and/or recoding censored values to missing can bias estimation parameters, influence variance estimates, and, in turn, yield questionable confidence intervals and test statistics. While these changes in parameters are relatively small in our analyses, our sample sizes are large, and, even under an artificially-inflated LLOS, the percent of censoring is relatively low (<20%). Despite this, our results clearly demonstrate that the substitution approach changed the descriptive statistics and regression parameter estimates, and these effects varied by the substitution value. Substituting censored data points with 0.01 pg/mL introduced greater variability in estimates than substituting with half the LLOS (i.e., 4.95 pg/mL or 9.85 pg/mL). Our results also demonstrate that the deletion approach can result in considerable differences in descriptive statistics and correlation and parameter estimates when compared to censored data and conventional substitution approaches. The deletion approach erases any signal represented by the censored data, increasing the risk of potential biases. Due to these limitations, statistical experts do not recommend the use of substitution nor deletion approaches for any percentage of censoring (Helsel, 2006, 2012). Our findings demonstrate the effects of these different approaches while also illustrating the ease of implementation of the specialized approaches with salivary data.

### 4.1. Preliminary guidance

Based on our findings and understanding of censored data statistical approaches, we make the following recommendations for researchers using censored salivary biomeasure data.

In the initial stages of data analysis, investigators should examine the distribution of censored data across their independent variables. While some level of association is generally expected (assuming a relationship between the censored variable and the predictor), extreme clustering of censored data points across an independent variable should be considered when interpreting model results. For example, if 90% of censored CRP determinations were among females in our sample, this would have altered our interpretation of the sex effects in our models.

When calculating summary statistics, there are several important considerations that should inform the choice of approach, including the sample size, distribution of the data, and percentage of censoring. For larger sample sizes with normal/log-normal distributions, the MLE approach performs well. When using smaller datasets, the ROS and K-M methods are more appropriate. In our analyses, we made a log-normality assumption when implementing MLE to account for violations of distributional assumptions in our data. Despite these adjustments, and our large sample size, our findings show the impact of the skewed data on the MLE estimates which are consistently different than those generated under the other approaches. Also, results from our subsample analyses show the effect of reduced sample size on MLE descriptive statistics as these parameters deviated from those generated via the other approaches more so than seen in the analyses of the full sample. In contrast, our ROS and K-M descriptive statistics were similar to each other and more consistent with those derived from the conventional methods. Thus, even after implementing a log-normality assumption for our skewed data, estimates from the ROS and K-M approaches were more appropriate than those generated via MLE. We recommend biomeasure researchers using censored and skewed data perform similar comparisons when assessing descriptive statistics.

Researchers interested in correlation analyses with censored variables should use the Kendall’s τ or Spearman’s ρ approaches to estimate these relationships. While these approaches require an initial substitution step, the substitution value used does not affect the estimation of the coefficient because these methods estimate levels of association using the rank of the observations instead of the actual measurements. This makes the Kendall’s and Spearman’s approaches more appropriate for censored data with substituted values which represent partial data points rather than precise measurements. Unlike Kendall’s and Spearman’s correlation, the Pearson’s correlation uses all data points as if they are observed values. Pearson’s correlation also assumes a linear relation between the two variables of interest and normality of the data for testing purposes, both of which are not required by the Spearman’s or Kendall’s approaches. Our results show relatively large differences in Pearson’s correlation coefficients when comparing findings from the full sample to those in the 100-person subsample, while the Kendall’s and Spearman’s coefficients across these samples are relatively stable. These findings, which may reflect differences related to violations of Pearson’s model assumptions as well as sample size differences, further support the use of Spearman’s and Kendall’s correlations as the optimal approaches to assessing correlations with censored biomeasure data.

When implementing regression analyses with censored outcome data, the general Tobit regression is a flexible modeling approach for left- and right-censored outcomes. This approach follows a similar structure as general linear models and can be easily performed in common statistical packages (e.g., R, SAS, and Stata; see Supplemental Materials for details). Compared to the deletion approach, Tobit regression is preferred as it does not alter nor bias the underlying data. Similar to deletion, substitution approaches can introduce bias and change the nature and shape of the underlying data. Further, implementing substitution can negatively affect model fit. As seen in our analyses, the substitution of censored data points can create an artificial clustering of the data that is not resolved in model estimation and can be seen in residual plots. These problems with model fit were not observed in our Tobit regression models. Investigators choosing conventional, rather than censored regression approaches, should also consider how this affects parameter estimation for different types of independent variables (e.g., continuous vs. categorical). As seen in our results, variable type may introduce another aspect of variability into the parameter estimations from conventional approaches that is difficult to assess unless estimates are compared to the general Tobit regression. If investigators choose to implement regression analyses with various conventional and censored data approaches, traditional model comparison indices, such as AIC, BIC, and log-likelihood, cannot be used as the underlying data are different across models. We suggest reviewing model residual plots to examine model fit across the different approaches.

Finally, it is important for researchers to remember that the percent of censored data is critical to assessing the impact of censoring on model results. In many salivary bioscience studies, the percent of censored data is less than 10%- a level at which, at least in our models using large samples, introduces limited variability in the descriptive statistics and regression results regardless of the estimation approach employed. While this is reassuring, we recommend that these, easily implemented, specialized analytic approaches for censored data be more widely adopted by biomeasure researchers across all fields. If conventional approaches are employed, sensitivity analyses should be performed to compare results from conventional methods and censored data methods. If the level of censoring is very low (e.g., less than 5%), the results may be very similar across modeling approaches. If the estimations are widely different, we recommend using a censored data method as these approaches are more appropriate and do not rely on alterations to the underlying data.

### 4.2. Limitations

Our analyses and interpretations provide the foundation for a set of recommended guidelines for salivary bioscientists using censored data. However, there are several limitations regarding our analyses that highlight the need for additional research to fully develop best practices for the field.

There are many characteristics of our CRP data that should be considered when making inferences about the analytic choices, and the effects of these choices, presented in this paper. First, the range of our CRP data is very wide, and additional analyses are needed to assess how this may have affected the estimates generated by the conventional and specialized analysis approaches. Also, even after log transforming our CRP data (or using a log-normality assumption in the case of the Tobit regression), the normality of residuals was not fully achieved. While divergence from normality was not severe, these violations may have affected model parameters. This is a common problem encountered with biomeasure data that exhibit considerable skew. Additional research assessing how to best handle such skewed distributions is needed. Also, our sample size is very large, especially for salivary bioscience investigations. This likely helped minimize the effect of the censored data, and of the various analytic approaches employed, on our results. However, in general, the same patterns of findings were observed in our subsample analyses of 100 participants as those conducted with the full sample. Future analyses that further examine variations in sample size while adjusting the percentage of censoring are an important next step in this area of research. Finally, our results show relatively large changes in regression parameters when using a very low value (0.01 pg/mL) as the substitution concentration compared to half the LLOS (i.e., 4.95 pg/mL or 9.85 pg/mL). It is important to note, however, that the impact of the substitution value on model parameters is particularly large in our analyses because the LLOS for CRP is relatively high (9.9 pg/mL), making our substitution value of 0.01 pg/mL extreme relative to the observed values of CRP. Given that this effect will vary by the assay’s lower limit of measurement, if an investigator chooses a substitution approach, we recommend using a substitution value that is relative to the assay’s lower threshold, rather than a fixed value which may introduce more variability in parameter estimates, as we see in our analyses.

Furthermore, we limit our discussion to methods for handling censored data with a single measurement limit (the lowest level of measurement). Analytic approaches for data with multiple limits of censoring (i.e., both left and right censoring), and their application to salivary biomeasure data, merit a separate paper. We also limited our discussion to censoring in the outcome variable. Specialized approaches addressing censoring in independent variables are not well developed, particularly in the regression framework. Moreover, the biomarker- based literature regarding data visualization and model fitting approaches for left-censored data is limited. Therefore, the methods employed in this study are based on the current state of knowledge and provide a starting point for researchers interested in these issues.

Limitations specific to the general Tobit regression approach are also important to highlight. For example, general Tobit regression has limited utility when variables have high levels of censoring (e.g., > 50% censored data) or when sample sizes are small (e.g., less than 25–50 observations) (Gleit, 1985; Shumway et al., 2002). While these limitations do not affect our analyses, future researchers should consider them when deciding on a censored regression approach. The levels of censoring assessed in this paper (9% and 15%) are typical for commonly-examined salivary analytes in the biobehavioral literature. However, the application of specialized censored data analysis approaches for data with high levels of non-detects will be particularly important for investigators interested in biomeasures present at a low levels in saliva (e.g., some disease-specific antibodies) and analytes indexing rare exposures (e.g., salivary lead levels). The interpretation of results from the general Tobit regression approach is also dependent on meeting the model assumptions, including normality and homoscedasticity of residuals, linearity of relations between the continuous variables and the outcome, and the independence of observations. Similar to other linear regression approaches, general Tobit models also need to be assessed for influential cases and multicollinearity. Tobit models, and the parameters they estimate, are more vulnerable to violations of these assumptions. While briefly described, detailed discussions of evaluating model assumptions, outliers, and multicollinearity in censored data statistical methods should be the focus of future research. Additional research is also needed to assess the application of specialized censored data approaches to more complex modeling methods in salivary bioscience research, such as longitudinal and multilevel analyses. The longitudinal version of Tobit regression is a censored data approach that may be used to analyze censored salivary biomeasure data that include multiple saliva sample collections per participant (e.g., diurnal sampling).

## 5. Conclusions

The censored data analysis methods examined in this paper produced findings that were generally similar with those generated via most conventional statistical analysis approaches, particularly when the percent of censoring was lower and the conventional approach substituted censored values with half the LLOS. The censored data approaches, however, required little to no manipulations to the underlying analyte data, and regression models using the specialized approaches had better fit than conventional approach models. Moreover, the censored data analyses were easy to implement, and their interpretation paralleled that of the conventional analytic approaches. Researchers using conventional approaches to analyze censored biomeasure data should explore the use of these censored data methods.

## Supplementary Material

Supplementary Material

## Figures and Tables

**Table 1:** Descriptive statistics for salivary c-reactive protein (CRP) concentrations in early adolescence using conventional and censored data approaches under two levels of censoring.

	Conventional Approaches			Censored Data Approaches^a^		
	Deletion	Substitution with ½ the LLOS	Substitution with 0.01 pg/mL	K-M	ROS	MLE

**True Level of Censoring (LLOS=9.9 pg/mL)–9% Left-censored**						
Mean	884.18	809.26	808.84	809.69	809.24	865.92
Median	149.60	119.01	119.01	118.54	119.01	118.36
Standard Deviation	3637.72	3487.69	3487.79	3487.85	3487.69	6275.62
**Artificially-inflated Level of Censoring (LLOS = 19.7 pg/mL)- 15% Left-censored**						
Mean	955.32	809.39	807.87	811.02	809.16	936.74
Median	179.88	119.01	119.01	118.54	119.01	112.73
Standard Deviation	3774.89	3487.66	3488.01	3487.80	3487.71	7727.19

Note: K-M = Kaplan-Meier, ROS = Regression on Order Statistics, MLE = Maximum Likelihood Estimation, LLOS = lower limit of sensitivity. Data and estimates are presented in their raw scale (pg/mL; not log-transformed). Descriptive statistics for the deletion approach under the true level of censoring (LLOS = 9.9 pg/mL) represent the observed CRP data.

Samples sizes: deletion approach with LL0S = 9.9 pg/mL: N = 569; deletion approach with LLOS = 19.7 pg/mL:N = 526; all other methods: N = 622.

a ROS and MLE estimates assume a log-normal distribution of CRP data and are subject to transformation bias. These estimation approaches require censored data points have a value; in these calculations censored values were recoded to ½ the LLOS

**Table 2:** Unadjusted associations between salivary c-reactive protein (CRP) and body mass index percentile score in early adolescence using conventional and censored data approaches under two levels of censoring.

	Conventional Approach Pearson’s r^a^	Rank-based Approaches^b^ Kendall’s τ	Censored Data Spearman’s ρ

**True Level of Censoring (LLOS=9.9 pg/mL))–9% Left-censored**			
Deletion	0.05	0.26***	0.37***
Substitution with ½ LLOS	0.06	0.28***	0.40***
Substitution with 0.01 pg/mL	0.06	0.28***	0.40***
**Artificially-inflated Level of Censoring (LLOS=19.7 pg/mL) – 15% Left-censored**			
Deletion	0.05	0.26***	0.37***
Substitution with ½ LLOS	0.06	0.28***	0.39***
Substitution with 0.01 pg/mL	0.06	0.28***	0.39***

Note: Deletion approach with LL0S = 9.9 pg/mL: N = 569, deletion approach with LLOS = 19.7 pg/mL: N = 526; all other methods: N = 622.

*** *p* < 0.001

a The Pearson’s correlation measures linear associations and makes bivariate normality assumptions. These assumptions may not be appropriate for these relations.

b The Spearman’s and Kendall’s rank-based correlations can be considered specialized methods for censored data. These estimation approaches require censored data points have a value, and these calculations recode censored values to either half the LLOS or 0.01 pg/mL. Once substituted with a value, all censored data points are ranked at the same level, making correlation coefficient estimates the same under both substitution approaches.

**Table 3:** Adjusted associations between salivary c-reactive protein (CRP) and body mass index (BMI) percentile score in early adolescence using conventional and censored data linear regression approaches under two levels of censoring.

	Conventional Approaches			Censored Data Approach Log-Normal Tobit
	Deletion	Substitution with ½ the LLOS	Substitution with 0.01 pg/mL	

**True Level of Censoring (LLOS=9.9 pg/mL)– 9% Left-censored**				
**Intercept (SD)**	3.70*** (0.25)	3.00*** (0.25)	1.37** (0.43)	2 92*** (0.26)
**BMI Percentile score (SD)**	0.02*** (0.00)	0.02*** (0.00)	0.03*** (0.00)	0.02*** (0.00)
**Female (SD)**	0.42** (0.13)	0.53*** (0.14)	0.86*** (0.24)	0.56*** (0.14)
**Very Good/Good Health (SD)**	−0.07 (0.17)	0.02 (0.18)	0.21 (0.31)	0.03 (0.19)
**Fair/Poor Health (SD)**	0.33 (0.24)	0.42^Δ^ (0.25)	0.63 (0.43)	0.43^Δ^ (0.26)
**N**	569	622	622	622
**Artificially-inflated Level of Censoring (LLOS=19.7 pg/mL)- 15% Left-censored**				
**Intercept (SD)**	4.03*** (0.24)	3.13*** (0.24)	0.40 (0.54)	2.94*** (0.26)
**BMI Percentile score (SD)**	0.01*** (0.00)	0.02*** (0.00)	0.04*** (0.01)	0.02*** (0.00)
**Female (SD)**	0.39** (0.13)	0.51*** (0.14)	0.98** (0.30)	0.55*** (0.14)
**Very Good/Good Health (SD)**	−0.08 (0.17)	0.01 (0.18)	0.18 (0.40)	0.01 (0.19)
**Fair/Poor Health (SD)**	0.39^Δ^ (0.23)	0.40 (0.25)	0.53 (0.54)	0.41 (0.26)
**N**	526	622	622	622

Note: Conventional approaches use log-transformed CRP values. Coefficients are all non-standardized and on the log scale. SD= Standard Deviation. Male and Excellent health are the reference categories.

* *p*<0.05

** *p* < 0.01.

*** *p* < 0.001

Δ *p* < 0.1.
