# Supplementary material for: Censored data considerations and analytical approaches for salivary bioscience data
Source: Psychoneuroendocrinology. Author manuscript; Available in PMC 2021 Jul 6. (PMC8260151; doi:10.1016/j.psyneuen.2021.105274)
Supplement: Supplementary Material [file NIHMS1711846-supplement-Supplementary_Material.docx]

**Supplementary Materials**

**Censored Data Considerations and Analytical Approaches for Salivary Bioscience Data**

**Specialized censored data approaches**

Methods such as Kendall’s Robust Line Fit (also known as Atkinson-Teil-Sen censored regression non-parametric line), Buckley-James multiple regression models, and the Cox proportion hazard models have limited applicability in salivary bioscience. Kendall’s Robust Line Fit can only be implemented using one outcome and one predictor variable. This approach has restricted utility in salivary bioscience studies which generally require adjustment for key covariates such as flow rate, time of day, and participant characteristics (e.g., oral health, age, and sex). Buckley-James multiple regression modeling was designed for, and has traditionally been used with, right-censored data (e.g., right censored survival data); therefore, its application in salivary bioscience is also limited. The Cox proportion hazard model can be used to estimate the proportional effects of one or more independent variables on a censored outcome. However, the interpretation of these effects, which translates to the probability of an analyte’s concentration falling below the assay threshold, has limited applicability to salivary bioscience research as most assay thresholds are set by manufacturers and do not hold conceptual nor physiological meaning.

**Tables**

Table S1. Descriptive statistics for salivary c-reactive protein (CRP) concentrations in early adolescence using conventional and censored data approaches under two levels of censoring for a subsample of 100 participants.

|  | **Conventional Approaches** | | | **Censored**  **Data Approaches**^a^ | | |
| --- | --- | --- | --- | --- | --- | --- |
|  | **Deletion** | **Substitution with ½ the LLOS** | **Substitution with**  **0.01 pg/mL** | **K-M** | **ROS** | **MLE** |
| **True Level of Censoring (LLOS=9.9 pg/mL)- 9% Left-censored** | | | | | | |
| Mean | 792.33 | 721.46 | 721.02 | 722.01 | 721.46 | 1296.84 |
| Median | 218.74 | 197.15 | 197.15 | 192.97 | 197.15 | 141.46 |
| Standard Deviation | 1396.82 | 1350.94 | 1351.17 | 1351.32 | 1350.94 | 11818.13 |
| **Artificially-inflated Level of Censoring (LLOS=19.7 pg/mL)- 15% Left-censored** | | | | | | |
| Mean | 847.28 | 721.66 | 720.19 | 723.29 | 721.48 | 1361.79 |
| Median | 225.60 | 197.15 | 197.15 | 192.97 | 197.15 | 136.94 |
| Standard Deviation | 1429.73 | 1350.83 | 1351.62 | 1351.17 | 1350.92 | 13473.60 |

Note: K-M= Kaplan-Meier, ROS= Regression on Order Statistics, MLE=Maximum Likelihood Estimation, LLOS= lower limit of sensitivity. Data and estimates are presented in their raw scale (pg/mL; not log-transformed). Descriptive statistics for the deletion approach under the true level of censoring (LLOS=9.9 pg/mL) represent the observed CRP data.

^a^ROS and MLE estimates assume a log-normal distribution of CRP data and are subject to transformation bias. These estimation approaches require censored data points have a value; in these calculations censored values were recoded to ½ the LLOS.

Samples sizes: deletion approach with LL0S=9.9 pg/mL: N=91; deletion approach with LLOS=19.7 pg/mL: N=85; all other methods: N=100.

Table S2. Unadjusted associations between salivary c-reactive protein (CRP) and body mass index percentile score in early adolescence using conventional and censored data approaches under two levels of censoring for a subsample of 100 participants.

|  | **Conventional Approach** | **Rank-based**  **Censored Data Approaches**^b^ | |
| --- | --- | --- | --- |
|  | **Pearson’s r**^a^ | **Kendall’s** $\boldsymbol{\tau}$ | **Spearman’s ρ** |
| **True Level of Censoring (LLOS=9.9 pg/mL) - 9% Left-censored** | | | |
| Deletion | 0.13 | 0.22^***^ | 0.32^***^ |
| Substitution with ½ LLOS | 0.15 | 0.25^***^ | 0.36^***^ |
| Substitution with 0.01 pg/mL | 0.15 | 0.25^***^ | 0.36^***^ |
| **Artificially-inflated Level of Censoring (LLOS=19.7 pg/mL) - 15% Left-censored** | | | |
| Deletion | 0.12 | 0.20^***^ | 0.29^***^ |
| Substitution with ½ LLOS | 0.15 | 0.25^***^ | 0.36^***^ |
| Substitution with 0.01 pg/mL | 0.15 | 0.25^***^ | 0.36^***^ |

Note: Deletion approach with LLOS= 9.9 pg/mL: N=91; deletion approach with LLOS=19.7 pg/mL: N=85; all other methods: N=100.

****p*<0.001.

^a^The Pearson’s correlation measures linear associations and makes bivariate normality assumptions. These assumptions may not be appropriate for these relations. ^b^The Spearman’s and Kendall’s rank-based correlations can be considered specialized methods for censored data. These estimation approaches require censored data points have a value, and these calculations recode censored values to either half the LLOS or 0.01 pg/mL. Once substituted with a value, all censored data points are ranked at the same level, making correlation coefficient estimates the same under both substitution approaches.

Table S3. Adjusted associations between salivary c-reactive protein (CRP) and body mass index (BMI) percentile score in early adolescence using conventional and censored data linear regression approaches under two levels of censoring for a subsample of 100 participants.

|  | **Conventional Approaches** | | | **Censored**  **Data Approach** |
| --- | --- | --- | --- | --- |
|  | **Deletion** | **Substitution with ½ the LLOS** | **Substitution with**  **0.01 pg/mL** | **Log-Normal Tobit** |
| **True Level of Censoring (LLOS=9.9 pg/mL)- 9% Left-censored** | | | | |
| **Intercept**  **(SD)** | 3.75***  (0.64) | 2.95***  (0.00) | 1.43  (1.21) | 2.88***  (0.70) |
| **BMI percentile score**  **(SD)** | 0.01  (0.02) | 0.01 ^Δ^  (0.01) | 0.02  (0.01) | 0.02 ^Δ^  (0.01) |
| **Female**  **(SD)** | 0.90*  (0.35) | 0.89 *  (0.38) | 0.99  (0.66) | 0.91*  (0.38) |
| **Very Good/Good Health**  **(SD)** | 0.19  (0.49) | 0.48  (0.52) | 1.03  (0.90) | 0.51  (0.52) |
| **Fair/Poor Health**  **(SD)** | 0.81  (0.65) | 0.87  (0.67) | 0.96  (1.17) | 0.87  (0.68) |
| **N** | 91 | 100 | 100 | 100 |
| **Artificially-inflated Level of Censoring (LLOS=19.7 pg/mL)- 15% Left-censored** | | | | |
| **Intercept**  **(SD)** | 4.12***  (0.63) | 3.07***  (0.6674) | 0.36  (1.43) | 2.91***  (0.70) |
| **BMI percentile score**  **(SD)** | 0.01  (0.01) | 0.01^Δ^  (0.01) | 0.03^Δ^  (0.02) | 0.02 ^Δ^  (0.01) |
| **Female**  **(SD)** | 0.77*  (0.34) | 0.89*  (0.36) | 1.38^Δ^  (0.78) | 0.93*  (0.38) |
| **Very Good/Good Health**  **(SD)** | 0.27  (0.48) | 0.42  (0.50) | 0.83  (1.07) | 0.45  (0.52) |
| **Fair/Poor Health**  **(SD)** | 0.73  (0.62) | 0.89  (0.65) | 1.22  (1.39) | 0.90  (0.68) |
| **N** | 85 | 100 | 100 | 100 |

Note: Conventional approaches use log-transformed CRP values. Coefficients are all non-standardized and on the log scale. SD= Standard Deviation. Male and Excellent health are the reference categories.

^Δ^*p*<0.1, **p*<0.05, ***p*<0.01, ****p*<0.001

**Figures**

Figure A.1. The distribution of salivary c-reactive protein (CRP) concentrations in early adolescence using the raw (left) and log-transformed (right) data. Panels A and B use the conventional deletion approach, and panels C and D use specialized censored data visualization approaches.

**
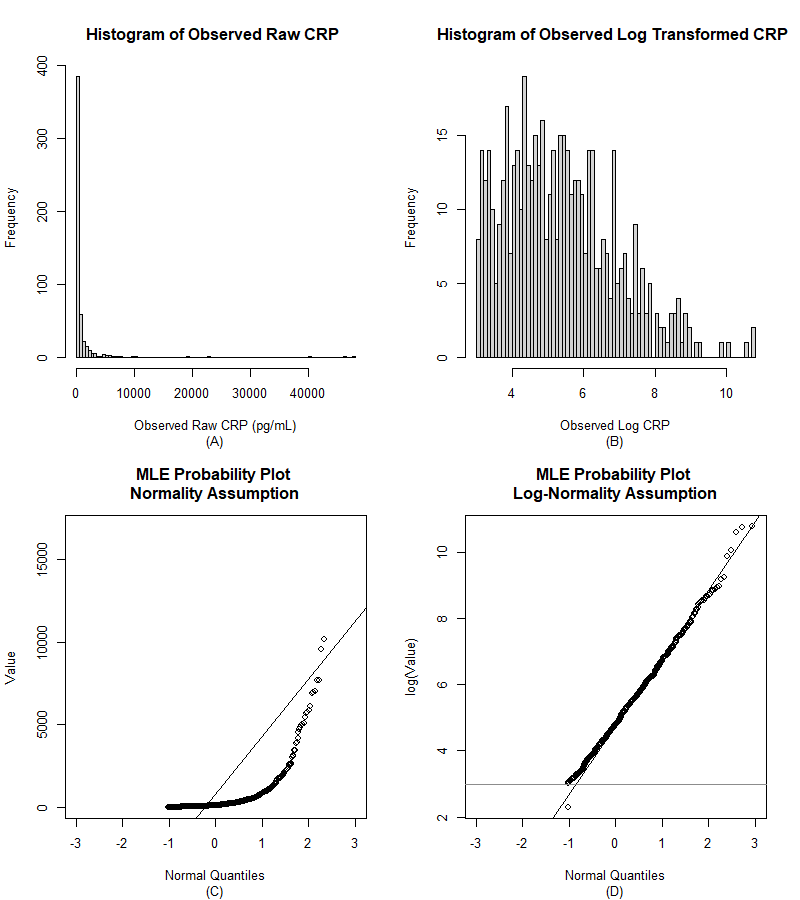
**

Note: N=526 for panel A and B; N=622 for panel C and D. These data are censored at the artificially-inflated lower limit of assay sensitivity (LLOS=19.7 pg/mL). Panel C is a typical Q-Q plot with a normality assumption for right skewed data (i.e., concave up) showing the non-normal distribution of the salivary CRP data. Panel D assumes log-normality and shows strong improvement in the distribution of the data under this assumption. The horizontal grey line is drawn at the log of the LLOS (ln(19.7)). The point below this line in panel D represents the censored values.

Figure A.2. Censored data scatter plots for raw (left) and log-transformed (right) salivary c-reactive protein (CRP) determinations in early adolescents (N=622) showed no systematic pattern of censoring across body mass index (BMI) percentile score.

**
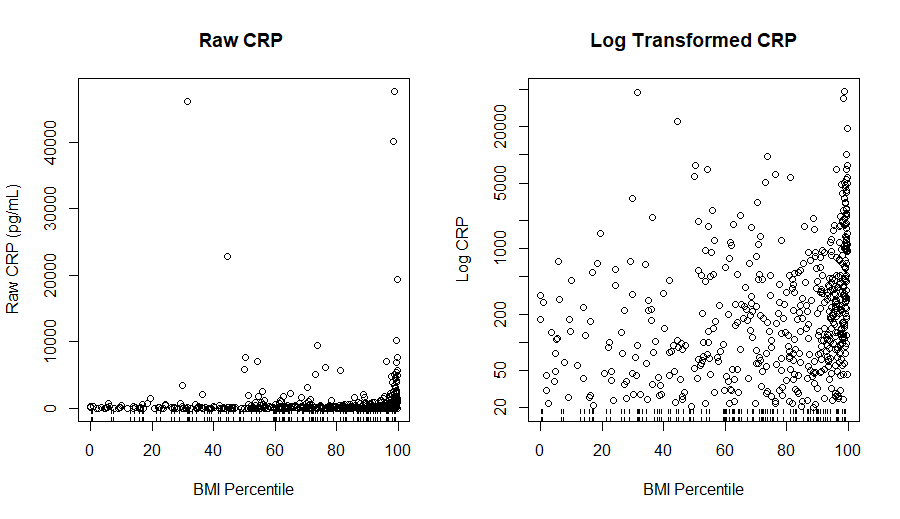
**

Note: The average BMI percentile score for participants with censored CRP determinations was 61.39 (median=65.39; range=0.26 - 99.25), and the average BMI percentile score for early adolescents with observed CRP determinations was 75.65 (median= 86.74; range=0.00 - 99.78).

Observed determinations of salivary CRP are plotted as individual points. The censored determinations of salivary CRP are represented by dashed lines spanning from zero to the LLOS. These data are censored at the artificially-inflated lower limit of assay sensitivity (LLOS=19.7 pg/mL).

Figure A.3. Censored data boxplots for raw (left) and log-transformed (right) salivary c-reactive protein (CRP) determinations across participant sex under two levels of left-censoring (N=622).

**
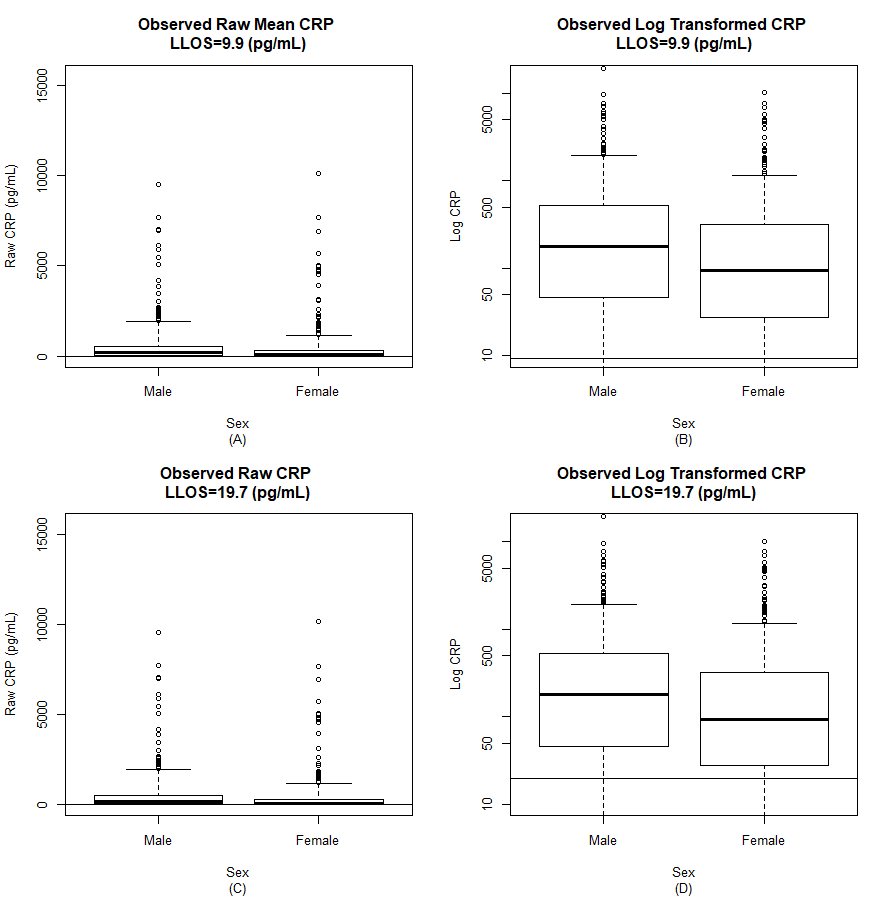
**

Note: Censoring thresholds are shown as horizontal lines. Regression on Order Statistic (ROS) was used to estimate the censored portion of the plot.

Figure A.4. Censored data boxplots for raw (left) and log-transformed (right) salivary c-reactive protein (CRP) determinations across participant health status under two levels of left-censoring (N=622).

**
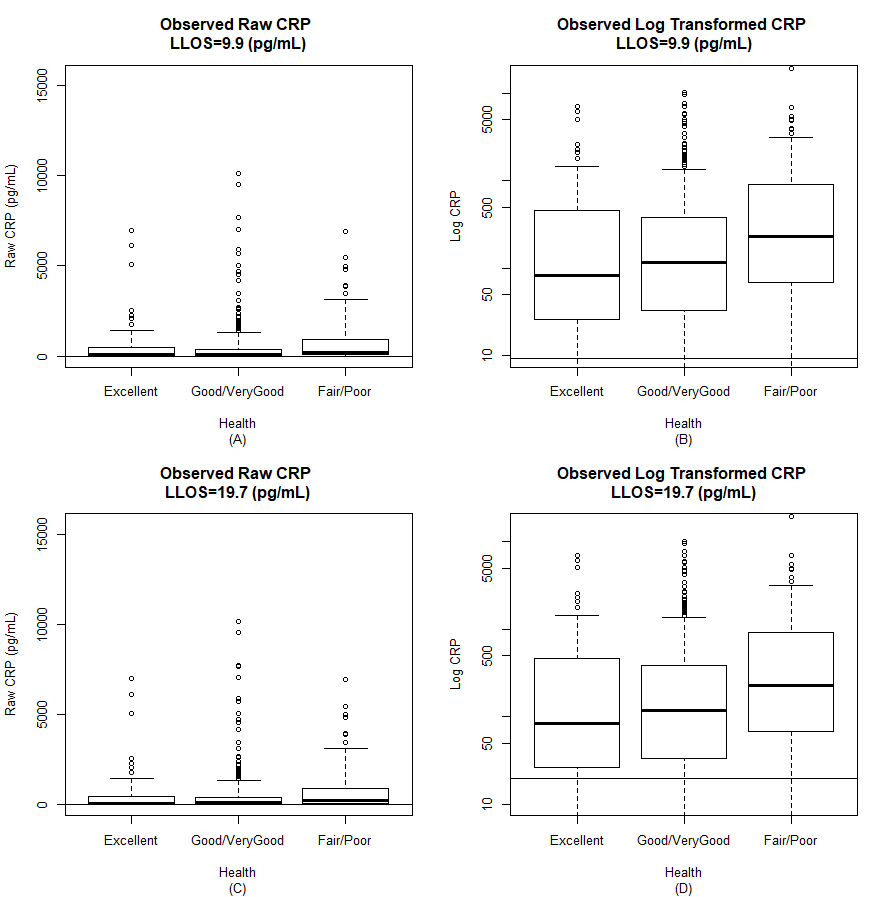
**

Note: Censoring thresholds are shown as horizontal lines. Regression on Order Statistic (ROS) was used to estimate the censored portion of the plot.

**Software Information**

Tobit regression is readily available in R, SAS, and Stata software packages. Currently, there is no procedure for Tobit analysis in SPSS; however, this may be developed in the future. SPSS offers an extension command that allows submission of R commands to the R package AER, namely SPSSINC TOBIT REGR. This can be accessed in the SPSS Developer Central Website Downloads section.

The log-likelihood of Tobit()(Kleiber and Zeileis, 2020) command in R uses the log-density $log(f(y_{i}))$ of the uncensored observations (by default: $y_{i}>0$) and the log-probability $log(F(c))$ of the censored observations (by default: $y_{i} = 0$). For heteroscedastic Tobit models, the package crch (Messner et al., 2019) is available for censored regression with conditional heteroscedasticity. It can also fit the classical Tobit model with additional functionality and options.

**References**

Kleiber, C., & Zeileis, A. (2020). AER: Applied Econometrics with R (Version 1.2-9) [Computer software]. <https://CRAN.R-project.org/package=AER>

Messner, J., Zeileis, A., & Stauffer, R. (2019). crch: Censored Regression with Conditional Heteroscedasticity (Version 1.0-4) [Computer software]. <https://CRAN.R-project.org/package=crch>
